# Midterm prognosis following total correction of tetralogy of fallot in adult patients

**DOI:** 10.3389/fcvm.2023.1254022

**Published:** 2023-10-16

**Authors:** Zahra Khajali, Nasibeh Mohammadi, Yaser Toloueitabar, Majid Maleki, Sedigheh Saedi, Zeinab nourouzi, Saeideh Mazloum-Zadeh, Maryam Chenaghloo, Amirhossein Jalali, Hassan Tatari, Maryam Aliramezany

**Affiliations:** ^1^ Rajaei Cardiovascular, Medical and Research Center, Iran University of Medical Sciences, Tehran, Iran; ^2^ Department of Cardiology, Zanjan University of Medical Sciences, Zanjan, Iran; ^3^ Cardiovascular Research Center, Tabriz University of Medical Sciences, Tabriz, Iran; ^4^ Cardiovascular Research Center, Institute of Basic and Clinical Physiology Sciences, Kerman University of Medical Sciences, Kerman, Iran

**Keywords:** tetralogy of fallot (TOF), tetralogy of fallot total correction (TFTC), late mortality, pulmonary valve replacement, adulthood

## Abstract

**Background:**

Tetralogy of Fallot is a common congenital heart disease characterized by cyanosis. The primary treatment approach involves corrective surgery typically performed within the first year of life to achieve complete resolution. However, certain patients may undergo surgery at an older age. This study seeks to assess the efficacy of surgery by examining the midterm outcomes of total correction of Tetralogy of Fallot when performed in older individuals.

**Methods:**

This interventional-longitudinal study focused on patients who underwent complete surgery to correct tetralogy of Fallot at an advanced age of over 15 years. All of the participants were referred to the Shahid Rajaei Heart and Vascular Center, which is a referral center for congenital heart diseases in Iran, between 2010 and 2020. The surgical procedures for these patients involved primary total correction of tetralogy of Fallot or surgery following by shunt implantation. Prior to the surgery, the necessary information was gathered from the patients' medical records. The patients were then monitored over a 5-year period, during which they received regular check-ups from cardiologist with fellowship in adult congenital heart disease.

**Results:**

A total of 94 participants were enrolled in the study, with an average age of 26.7 ± 9.6 years. Notably, the majority of the participants were male. The study reported a late mortality rate of 3.2%. Furthermore, 17 patients, constituting 18% of the cohort, underwent a secondary surgical procedure. This secondary surgery encompassed 14 cases of Pulmonary Valve Replacement (14.8%) and 3 cases of Ventricular Septal Defect repair (3.1%).

**Conclusion:**

While the optimal age for total correction of Tetralogy of Fallot is conventionally considered to be within the first year of life, this study demonstrated that surgical intervention performed at a later stage of life can yield favorable midterm prognoses. It is imperative to emphasize that individuals unable to undergo surgery at the ideal age due to a multitude of factors should not be deprived of the potential benefits associated with surgical intervention.

## Background

Tetralogy of Fallot is a prevalent congenital heart disease characterized by cyanosis, with an incidence rate of 0.34 per 1,000 live births (1). The fundamental tetrad of abnormalities defining Tetralogy of Fallot was initially elucidated by Bishop and anatomist Nicolas Steno in 1673 (2). Subsequently, the comprehensive anatomical understanding of this condition was provided by the French physician Étienne-Louis Fallot in 1888 (3). The surgical approach required for individuals with Tetralogy of Fallot is contingent upon the specific anatomical characteristics of the condition and the clinical manifestations exhibited by the patient (2). Furthermore, the extent of right ventricular outflow tract stenosis can contribute to varying degrees of cyanosis in Tetralogy of Fallot patients (4). Consequently, in certain cases, the diagnosis and surgical correction of the disease may be postponed until adulthood (5). The primary therapeutic approach for Tetralogy of Fallot involves total correction through surgical intervention. This surgical method was originally delineated by Lillehei et al. in 1955 (6). However, as previously elucidated, there are scenarios where the surgical correction of Tetralogy of Fallot may encounter delays due to diverse reasons. In such circumstances, patients may contend with prolonged cyanosis and hypoxia, leading to enduring physiological consequences. Furthermore, the right ventricular pressure overload can precipitate various systemic complications encompassing polycythemia, renal issues, arrhythmias, and neurological complications. These alterations in physiological and hemodynamic parameters can exert a substantial influence on both the surgical procedure's efficacy and its ultimate outcomes (7, 8).

The ideal timeframe for surgical intervention in Tetralogy of Fallot is within the inaugural year of life, a window of opportunity correlated with enhanced prognoses and diminished risk of complications (2). It is worth noting that surgeries conducted at the ages of 3, 6, and 11 months are also deemed acceptable and are supported by clinical recommendations (9–11).

### Study objective

Given the possible controversies surrounding and the potential delays in the total correction of Tetralogy of Fallot, there exists a compelling need to comprehensively scrutinize the outcomes of surgical procedures conducted at an advanced age. In light of this, the primary objective of our study is to meticulously investigate and analyze the midterm complications stemming from complete surgical correction of Tetralogy of Fallot in patients aged over 15 years.

## Methods

### Design

This study represents an interventional-longitudinal investigation centered on individuals who underwent total correction surgery for Tetralogy of Fallot at an advanced age, surpassing 15 years (1). The entire cohort of participants was referred to the Shahid Rajaei Heart and Vascular Center, recognized as a premier referral facility for congenital heart diseases in Iran, spanning the timeframe from 2010 to 2020. The surgical procedures administered to these patients encompassed primary total correction of Tetralogy of Fallot in 78 cases, while in 16 patients (17.02%), total correction was performed subsequent to shunt implantation. Notably, a consistent surgical team managed the treatment of all patients. Given that a significant portion of these individuals had previously undergone shunt implantation during their early years, the surgical approach employed for this subgroup involved posterolateral thoracotomy. Additionally, the continuity of care was ensured by assigning the same cardiologist, specialized in adult congenital heart disease through fellowship training, to oversee the patients' medical management both preoperatively and postoperatively. Prior to the surgical interventions, comprehensive patient data was meticulously collated from their medical records, encompassing pertinent diagnostic information such as echocardiographic findings and angiographic or CT angiographic results The preoperative evaluation of the majority of our patients relied on CT angiography, a method that afforded a comprehensive assessment of pulmonary branch and Major Aortopulmonary Collateral Arteries (MAPCAs) dimensions, along with coronary artery examination. In cases involving older patients or necessitating more extensive coronary artery assessment due to elevated pulmonary pressures, cardiac catheterization was performed.

Following surgery, a systematic follow-up protocol was instituted, with patients scheduled for regular visits at intervals ranging from 3 to 6 months, contingent on symptomatology and disease severity, spanning a 5-year period. Each follow-up appointment entailed a comprehensive review of medical history, physical examination, and transthoracic echocardiography. In instances where echocardiographic and CT angiographic findings were incongruent, further assessment via cardiac magnetic resonance imaging (CMR) was carried out. Notably, routine brain scans were not administered to all patients but were selectively conducted when patients exhibited symptoms or were suspected of having brain lesions. It is pertinent to mention that this aspect was not included in the compiled dataset. Throughout the follow-up period, vigilant monitoring was upheld to track instances of late mortality and the necessity for secondary surgeries, with all pertinent occurrences diligently documented in a checklist.

### Surgical technique

Following median sternotomy and heparin administration, cannulation was initiated for the aorta, superior vena cava (SVC), and inferior vena cava (IVC) to initiate cardiopulmonary bypass (CPB). Hypothermia was induced, typically reaching 30°C, although deeper hypothermia was employed when there was a significant blood return from the left atrium. In cases where a Modified Blalock Taussig shunt was present, it was closed, and cardiac arrest was induced by administering del Nido cardioplegic solution into the aortic root at 60-min intervals. Closure of the ventricular septal defect (VSD) was performed via the right atrial approach, utilizing a polytetrafluoroethylene (PTFE) patch and interrupted pledgeted proline sutures. Refinement of the right ventricular outflow tract (RVOT) was achieved either through a longitudinal incision on the RVOT or via the tricuspid valve, depending on the individual case.

The main pulmonary artery (PA) was longitudinally opened, and a commissurotomy of the pulmonary valve was conducted. The dimension of the pulmonary valve annulus was meticulously assessed, and in cases where it was determined to be undersized (typically indicated by a Z score around −2), a pulmonary valve replacement procedure was undertaken, employing a biologic prosthetic valve. Additionally, the right ventricular outflow tract (RVOT) and the main pulmonary artery (PA) received augmentation through the application of an autologous pericardial patch, with additional PA branch augmentation performed in instances of stenosis. In all patients requiring valve replacement, a biologic pulmonary valve with a size ranging between 23 and 25 was employed. The remaining steps of the operation followed the standard protocol, and the patient was gradually weaned off cardiopulmonary bypass (CPB) after the rewarming process. In cases where doubts persisted regarding the presence of residual pulmonary stenosis, right ventricular pressure was assessed using a transducer. A right-to-left ventricular pressure ratio of less than 0.7 was established as the acceptable threshold. For the majority of patients, the management of large MAPCAs involved preoperative closure using coils. During surgery, if the surgeon had the necessary access, further closure of MAPCAs was undertaken. In a few instances post-surgery, patients experienced hemoptysis from MAPCAs, necessitating their subsequent closure using coils.

### Inclusion criteria

The study included all individuals who underwent surgery for tetralogy of Fallot at our facility when they were 15 years old or above.

### Exclusion criteria

Patients whose information was incomplete were not included in this study.

### Instrument

The information extracted from the medical records, as well as the data collected during the 5-year follow-up period and recorded in the checklist, were divided into four distinct groups:

### Demographic data

This included the age at the time of surgery, gender, and other related demographic information.

### Paraclinical data

This category encompassed a range of diagnostic evaluations and their respective findings, including:

• Electrocardiography, used for identifying cardiac arrhythmias.
• Echocardiography, offering insights into various parameters such as the ejection fraction of both the right and left ventricles, the severity of tricuspid and aortic regurgitation, pulmonary branch size (defined as small when the Mac Goon's ratio was <1.2), and the degree of right ventricular outflow tract (RVOT) obstruction.
• CT angiography, employed to detect the presence of major aorto-pulmonary collateral arteries (MAPCAs) and any additional anomalies, such as Atrial Septal Defect (ASD), Transposition of the Great Arteries (TGA), Patent Ductus Arteriosus (PDA), or coronary anomalies.
• Cardiac Magnetic Resonance Imaging (CMR), which provided supplementary diagnostic information.
• Requisite laboratory data as needed.

Comprehensive laboratory tests were conducted for all patients as a prerequisite before the surgical procedures. In cases where patients exhibited symptoms or presented with elevated hematocrit levels, phlebotomy was executed following the administration of iron supplements, if deemed necessary. Moreover, if deemed necessary, patients received platelet transfusions prior to surgery.

### Surgery information

Within this category, data pertaining to the application of the Right Ventricular Outflow Tract (RVOT) patch during surgery were documented. The “Five-year outcome” group encompassed information regarding instances of late-onset mortality, defined as events occurring 6 months or more following surgery, as well as the requirement for subsequent surgeries, including pulmonary valve replacement or residual Ventricular Septal Defect (VSD) repair.

### Data analysis

Data were analyzed using descriptive statistics and chi square through SPSS 22.

## Results

This study encompassed a cohort of 94 patients, who were admitted to the Rajai Cardiovascular, Medical, and Research Center between 2010 and 2020. These patients, aged over 15 years, were candidates for complete correction of Tetralogy of Fallot and underwent a comprehensive evaluation. A rigorous follow-up protocol spanning 5 years was established to monitor their progress.

At the time of surgical intervention, the patients exhibited a mean age of 26.7 ± 9.6 years, with a notable majority being male, constituting 59.6% of the total cohort. It's noteworthy that the average age of male patients was significantly higher than that of female patients (*P*-value: 0.009). Additionally, 27.7% of the patients presented with associated anomalies. Further demographic details are provided in Table 1. Notably, the size of pulmonary branches before surgery met acceptable criteria in 77 individuals (81.9%).

**Table 1:** Demographic data of patients.

Variables of demographic data	Frequency (percent)	*P*-value
Age		
Mean	26.7 ± 9.6 years	
Age groups		0.938
15–25	52 (55.3%)	
26–35	27 (28.7%)	
36–45	13 (13.8%)	
46–55	2 (2.1%)	
Gender		**0**.**009**
Male	56 (59.6%)	** **
Female	38 (40.4%)	** **
Cardiac anomalies		0.024
Yes	26 (27.7%)	** **
No	68 (72.3%)	** **
MAPCA (major aortopulmonary collateral arteries)		0.203
Yes	38 (40.4%)	
No	56 (59.6%)	** **
Arrhythmia		0.205
Yes	17 (18.1%)	
No	77 (81.9%)	
Sidedness		0.216
Right	24 (25.5%)	
Left	70 (74.5%)	
RVOT patch	91 (96.8%)	

Bold value shows the difference is significant at *P* < 0.05.

Furthermore, in 91 patients (96.8%), a Right Ventricular Outflow Tract (RVOT) patch was employed during surgery. Postoperatively, RVOT obstruction was successfully resolved in 50.5% of the patients. Detailed information regarding right ventricular outflow tract obstruction before and after surgery is presented in Table 2. Notably, a small or hypoplastic pulmonary artery was identified in 17 patients (18.1%), with these figures changing to 91 (96.8%) and 3 (3.2%) after surgery, respectively. Statistical analysis did not reveal any significant differences in right ventricular (*P*-value: 0.338) or left ventricular (*P*-value: 0.062) function before and after the surgical intervention.

**Table 2:** RVOT characteristics before and after surgery.

RVOT obstruction	Before surgery	After surgery
Mild	2.1%	39.3%
Moderate	3.1%	12.7%
Severe	94.6%	0%

This study evaluated the 5-year outcomes of 94 patients who underwent total correction surgery for Tetralogy of Fallot at the Rajai Cardiovascular, Medical, and Research Center between 2010 and 2020. The recorded late mortality rate was 3.2%, and 17 patients (18%) necessitated a secondary surgical procedure. Among these, 14 procedures (14.8%) involved Pulmonary Valve Replacement (PVR), and 3 (3.1%) entailed Ventricular Septal Defect (VSD) closures. The indication for a second surgery in three patients stemmed from an unstable condition attributed to the detachment of VSD patches.

In total, 31 patients (32.9%) underwent Pulmonary Valve Replacement (PVR), with 17 patients (18%) receiving this intervention during the initial surgery and 14 patients (14.8%) requiring it during a subsequent surgical procedure (refer to Table 3). Notably, the study's analysis identified age as the sole factor significantly influencing the necessity for a second surgery. Within the 26–35-year age group, there was a substantial increase in the demand for a second surgery, a finding that exhibited statistical significance (*p*-value: 0.03). Within this age bracket, 37% of patients necessitated re-surgery during the 5-year follow-up period, with 9 patients undergoing PVR and 1 patient requiring residual Ventricular Septal Defect (VSD) repair. Interestingly, gender, the presence of a history of cardiac anomalies, arrhythmias, or Major Aortopulmonary Collateral Arteries (MAPCAs) did not yield any statistically significant effects on late mortality according to the study's findings. Among the patients, a noteworthy complication observed in this cohort was hemoptysis originating from the Major Aortopulmonary Collateral Arteries (MAPCAs) post-surgery, which manifested in two cases. These complications were effectively managed through interventional procedures, ultimately leading to the successful closure of the affected vessels.

**Table 3:** Late outcomes of surgery.

Variable	Frequency (percent)
Needing for the second surgery	17 (18.1%)
PVR (pulmonary valve replacement)	14 (14.9%)
VSD (ventricular septal defect)	3 (3.2%)
Late mortality	3 (3.2%)

## Discussion

The primary objective of this study was to investigate the complications, mortality rates, and midterm prognosis in patients diagnosed with Tetralogy of Fallot who underwent total correction surgery at an advanced age. This investigation is prompted by ongoing debates regarding the optimal timing for corrective surgery in this condition. Notably, the average age of our patient cohort at the time of surgery stood at 26.7 ± 9.6 years, with a higher prevalence of male patients, consistent with the demographic distribution observed in the majority of prior studies (5, 12–14).

Previous studies have indicated that estrogen hormone may exert protective effects on ventricular function (2). Furthermore, it is well-established that men typically exhibit larger right and left ventricular volumes and sizes, attributed to their increased body mass compared to women. These physiological differences may account for the higher prevalence of male patients in our study. These patients, due to the mentioned factors, may experience more pronounced symptoms, prompting them to seek medical attention.

It is noteworthy that a study conducted by Atik et al. on a comparable patient population reported a similar average age at surgery of 26.6 ± 11.1 years (14). The variation in patient age at the time of surgery across different studies may potentially be attributed to factors such as delayed disease diagnosis, limited diagnostic capabilities in less developed regions, or patients' lack of awareness regarding the necessity for timely surgical intervention.

In our study, the observed prevalence rates of right isomerism and arrhythmia were 25.5% and 18.1%, respectively. Notably, these rates appear to be lower in comparison to the figures reported in prior studies within this domain (12, 14–16). Such disparities may signify the inherent heterogeneity among Tetralogy of Fallot patients. Conversely, our patient cohort exhibited a higher prevalence of cardiac anomalies and Major Aortopulmonary Collateral Arteries (MAPCAs), with 40.4% of patients presenting with MAPCAs. This finding contrasts with the results reported in another study (5, 16), which documented lower incidences of these conditions. It is conceivable that the variance in findings could be attributed, at least in part, to the older age of patients included in our study.

The present study identified a late mortality rate of 3.2%, a figure consistent with the range of rates documented in earlier research, which typically falls between 2.5% and 7.7% (12–14). Notably, the majority of fatalities occurred approximately 6 months following surgery and were primarily attributed to sudden cardiac death, presumably linked to arrhythmias, with one exception being attributed to severe right ventricular failure. Sudden cardiac death can be precipitated by arrhythmias, a condition more prevalent in cases characterized by prolonged disease duration and right ventricular pressure overload, ultimately culminating in ventricular failure

Studies conducted on cases where corrective surgery was performed at an earlier age consistently report a lower incidence of late mortality. As an illustration, in a study led by Hyunqtae Kim et al., with an average patient age at the time of surgery of 13 months, the recorded late mortality rate stood at 2.5%, while the survival rate was notably high at 97% (17). Overall, it can be deduced that individuals who have undergone complete correction surgery during childhood generally exhibit favorable long-term survival and clinical outcomes (18), boasting a reported survival rate ranging from 85% to 90% (9).

By comparing findings across multiple studies, it becomes evident that late mortality in patients who undergo surgery at an older age tends to be somewhat elevated compared to those who undergo surgery at a younger age. One of the pivotal contributing factors to this divergence likely stems from the prolonged disease duration. As the patient's age at the time of surgery increases, the duration of exposure to hypoxia extends, and this chronic hypoxia, coupled with cyanosis, can lead to adverse remodeling of the right ventricle, compounded by systemic effects. Furthermore, the presence of right ventricular outflow tract stenosis and its associated complications emerges as a critical determinant influencing surgical outcomes.

In our study, the mean duration of Intensive Care Unit (ICU) admission was calculated at 87 ± 30 h. This outcome aligns with the results of previous research (5, 13), which also documented comparable mean ICU admission durations. It is worth noting, however, that Atik et al. reported a shorter ICU admission period in their investigation (14).

In our study, a second surgery was required by 18% of patients, with the majority undergoing Pulmonary Valve Replacement (PVR) at a rate of 14.9%, and a smaller fraction necessitating residual Ventricular Septal Defect (VSD) repair, accounting for 3.2% of cases. Among the patients who underwent PVR, pulmonary regurgitation (PR) was the predominant concern. In total, 31 patients (32.9%) underwent PVR, with 17 patients (18%) undergoing the procedure during the initial surgery and 14 patients (14.8%) during subsequent follow-up evaluations. It is noteworthy that in a study by Erdoğan et al., they reported that 6.2% of their patients required reoperation due to VSD, with some of these cases involving residual obstruction or pulmonary regurgitation (13).

The study conducted by Khalid et al. contributes additional empirical evidence bolstering the overall positive outcomes associated with surgical interventions aimed at achieving complete correction of Tetralogy of Fallot in older patients. The study's findings indicate that, while a minority of patients may exhibit residual ventricular septal defects following surgery, these defects tend to be of a mild nature and typically do not necessitate further surgical intervention. Specifically, the study reports a residual VSD rate of 8.7% among patients aged over 16 years, with none of these observed defects categorized as moderate or severe in severity.

These results underscore the critical importance of meticulous postoperative surveillance and the adept management of patients who have undergone Tetralogy of Fallot corrective surgery, thereby facilitating the timely identification and appropriate handling of any prospective complications. In summation, the outcomes reported in this study lend robust support to the favorable long-term prognostication associated with surgical interventions for Tetralogy of Fallot in older patient cohorts (5).

The study conducted by Atik et al. offers valuable insights into the outcomes of corrective surgery for Tetralogy of Fallot within the older population. The findings underscore that, while the overall requirement for re-surgery remains relatively low in this demographic, a minority of patients may still necessitate additional interventions during the follow-up period. To be specific, the study reported a 7.6% need for re-surgery among patients over a 45.1-month follow-up duration, with 2.5% of patients requiring pulmonary valve replacement and 5.1% undergoing VSD closure surgery.

These results emphasize the crucial significance of vigilant, long-term monitoring and comprehensive management of patients who have undergone corrective surgery for Tetralogy of Fallot, irrespective of age. Diligent follow-up care serves as a vital means to promptly identify and effectively address potential complications, thereby contributing to improved overall outcomes and reducing the likelihood of re-intervention (14). The study conducted by Chun Soo Park provides valuable insights into the outcomes of surgical interventions for Tetralogy of Fallot conducted at a younger age. The findings indicate that, while the mortality rate in this particular population remains relatively low, there exists a notable prevalence of surgery or re-intervention requirements during the follow-up period. Specifically, the study reported a mortality rate of 1.7% and a 31.7% need for surgery or re-intervention over the course of the follow-up period. These findings underscore the critical importance of implementing a comprehensive and individualized approach to postoperative care for patients who have undergone corrective surgery for Tetralogy of Fallot at a young age. Furthermore, these results suggest that, although surgery at a younger age can yield effective results, it may also entail a heightened risk of long-term complications necessitating additional interventions. Therefore, the meticulous monitoring and management of these patients stand as pivotal factors in achieving the most optimal outcomes (19).

The study conducted by Christos Alexiou contributes further substantiating evidence regarding the paramount importance of early intervention in Tetralogy of Fallot cases. The findings elucidate that, although the prognosis following complete correction surgery in patients at the age of 6 months generally yields favorable results, a minority may necessitate subsequent surgeries or re-intervention during the follow-up period. Notably, the occurrence of a single case of late non-cardiac-related mortality underscores the imperative need for comprehensive and individually tailored follow-up care for Tetralogy of Fallot patients, even following a successful corrective surgical procedure. In sum, these findings accentuate the critical significance of early diagnosis and timely intervention in realizing the most favorable outcomes for patients afflicted with Tetralogy of Fallot (20).

While the risk of complications following complete correction surgery for Tetralogy of Fallot in older patients may exhibit a slightly higher propensity in comparison to surgery performed at a younger age, both our study and analogous research endeavors have consistently demonstrated that surgical outcomes in the elderly remain within an acceptable range. Nevertheless, it is pivotal to underscore that complete correction conducted before the age of one year is considered inherently safe and yields substantial improvements in patient survival, relative to surgeries performed beyond this early window. Therefore, the importance of early diagnosis and intervention cannot be overstated, as they are pivotal in achieving the most favorable outcomes in Tetralogy of Fallot patients.

However, for those presenting later in life or unable to undergo surgery during infancy, corrective surgery at an older age can still represent a viable option with outcomes deemed acceptable. Nevertheless, stringent measures should be implemented to monitor and address potential complications (21).

The preservation of myocardial health stands as a fundamental aspect in Tetralogy of Fallot management, and early intervention through corrective surgery plays a pivotal role in averting abnormal right ventricular remodeling and hypertrophy. This, in turn, contributes to the reduction of ventricular arrhythmia risk and late all-cause mortality. Multiple studies have conclusively indicated that surgery performed at 1 year of age or younger yields superior myocardial preservation and outcomes when contrasted with surgeries undertaken later in life. Thus, the imperative of early diagnosis and intervention remains an essential cornerstone in optimizing outcomes for Tetralogy of Fallot patients.

Patients who underwent surgery for Tetralogy of Fallot correction at an age beyond the recommended timeframe demonstrated favorable outcomes from their surgery, suggesting that the procedure remains efficacious even when conducted in later stages of life (22, 23). While the optimal window for corrective surgery in Tetralogy of Fallot is conventionally recognized as being within the first year of life, it is important to acknowledge that patients may present at a later stage due to a variety of factors. Consequently, conducting a comprehensive anatomical and hemodynamic assessment before surgery becomes imperative to discern the most suitable surgical technique for each individual patient. Furthermore, the diagnosis and management of concomitant cardiac anomalies, hematological disorders, and arrhythmias can contribute significantly to the mitigation of adverse surgical outcomes. The regular and systematic monitoring of patients for clinical symptoms is of paramount importance in identifying those who may necessitate re-surgical intervention. Furthermore, as patients age, it is imperative to factor in the heightened risk of valvular diseases and coronary artery disease in the follow-up care of individuals who have undergone corrective surgery for Tetralogy of Fallot. In summation, the management of patients with Tetralogy of Fallot mandates a holistic and tailored approach to attain the most optimal outcomes.

However, our study is not without limitations. The primary challenge we encountered during its execution was the inherent heterogeneity among patients, both in terms of age and anatomical variations. This heterogeneity could potentially be mitigated through an expansion of the sample size. Moreover, over the course of the follow-up period, a subset of patients failed to adhere to regular visits, necessitating their exclusion from the study.

Furthermore, the study's design inherently precluded an in-depth exploration of the underlying mechanisms that might elucidate the observed outcomes. Therefore, future studies should be purposefully structured to provide mechanistic insights into the observed effects

## Conclusion

While the optimal age for achieving complete correction of Tetralogy of Fallot is conventionally considered to be within the first year of life, this study presents evidence indicating that surgical intervention at an advanced age can yield acceptable midterm outcomes. Notably, the foremost physiological transformation following Tetralogy of Fallot surgery involves the elimination of right ventricular outflow obstruction and the consequent resolution of cyanosis. Consequently, the risk of complications typically associated with cyanotic patients diminishes in these cases, which is anticipated to result in a reduction of symptomatology, hospitalization rates, and enhancement in patients' overall quality of life.

It is imperative to acknowledge that healthcare practitioners must remain cognizant of the possibility of delayed referrals for treatment in Tetralogy of Fallot patients, owing to various factors. Furthermore, it is critical to recognize that surgical outcomes remain satisfactory even in older age cohorts, exerting a potential influence on diverse facets of patients' lives. Consequently, the decision to pursue surgery should not be arbitrarily ruled out solely based on age considerations. In such scenarios, a regimen of systematic and frequent follow-up assessments is recommended to facilitate timely detection and management of any prospective complications. This approach serves to optimize surgical outcomes and mitigate the risk of adverse events in patients undergoing corrective surgery for Tetralogy of Fallot at an advanced age.

## Data Availability

The original contributions presented in the study are included in the article/Supplementary Material, further inquiries can be directed to the corresponding author.
